# Molecular and Biological Characterisation of *Turnip mosaic virus* Isolates Infecting Poppy (*Papaver* *somniferum* and *P. rhoeas*) in Slovakia

**DOI:** 10.3390/v10080430

**Published:** 2018-08-14

**Authors:** Miroslav Glasa, Katarína Šoltys, Lukáš Predajňa, Nina Sihelská, Slavomíra Nováková, Zdeno Šubr, Ján Kraic, Daniel Mihálik

**Affiliations:** 1 Institute of Virology, Biomedical Research Centre, Slovak Academy of Sciences, Dúbravská cesta 9, 845 05 Bratislava, Slovakia; Lukas.Predajna@savba.sk (L.P.); preninu@gmail.com (N.S.); Slavomira.Novakova@savba.sk (S.N.); Zdeno.Subr@savba.sk (Z.Š.); 2 Comenius University Science Park, Comenius University in Bratislava, Ilkovičova 8, 841 04 Bratislava, Slovakia; katarina.soltys@gmail.com; 3 Department of Biotechnologies, Faculty of Natural Sciences, University of Ss. Cyril and Methodius, J. Herdu 2, 917 01 Trnava, Slovakia; kraic@vurv.sk (J.K.); mihalik@vurv.sk (D.M.); 4 National Agriculture and Food Centre, Research Institute of Plant Production, Bratislavská cesta 122, 921 68 Piešťany, Slovakia; 5 Institute of High Mountain Biology, University of Žilina, Univerzitná 8215/1, 010 26 Žilina, Slovakia

**Keywords:** *Papaveraceae*, TuMV, genome, recombination, HTS, symptoms

## Abstract

In recent years, the accumulated molecular data of *Turnip mosaic virus* (TuMV) isolates from various hosts originating from different parts of the world considerably helped to understand the genetic complexity and evolutionary history of the virus. In this work, four complete TuMV genomes (HC9, PK1, MS04, MS15) were characterised from naturally infected cultivated and wild-growing *Papaver* spp., hosts from which only very scarce data were available previously. Phylogenetic analyses showed the affiliation of Slovak *Papaver* isolates to the world-B and basal-B groups. The PK1 isolate showed a novel intra-lineage recombination pattern, further confirming the important role of recombination in the shaping of TuMV genetic diversity. Biological assays indicated that the intensity of symptoms in experimentally inoculated oilseed poppy are correlated to TuMV accumulation level in leaves. This is the first report of TuMV in poppy plants in Slovakia.

## 1. Introduction

The *Papaver* genus in the *Papaveraceae* family comprises up to 100 plant species distributed in various areas of the world. Among them, viral infection of two *Papaver* species was investigated in the present study, i.e., oilseed poppy (*P. somniferum* L.) and common poppy (*P. rhoeas* L.). Originating from Asia Minor or from the Western Mediterranean region, oilseed poppy cultivation covers a wide geographic area around the world due to the adaption of this species to various climatic conditions as a result of its long history of domestication and breeding. The oilseed poppy is legally grown in Slovakia for the seeds used as an ingredient in some foods and for the seed capsules containing several alkaloids, including morphine, codeine, papaverine, and thebaine, valuable in the pharmaceutical industry [1,2,3]. On the contrary, common poppy is a red-flowering agricultural weed abundantly spread in Europe [4].

Few viruses infecting these *Papaver* spp. have been reported to date [5]. Viruses known from older records to infect these species include *Bean yellow mosaic virus* (BYMV), *Beet mosaic virus* (BtMV) and *Turnip mosaic virus* (TuMV) from the genus *Potyvirus*, and a polerovirus, *Beet western yellows virus* (BWYV) [5,6]. More recently, a new umbravirus, *Opium poppy mosaic virus*, was identified as causing leaf mosaic and mottling in opium poppy in New Zealand [7] and *Tomato leaf curl New Delhi virus* was detected from poppies showing curling of leaves in India [8]. Despite these reports, knowledge of viral pathogens naturally infecting poppy remains limited.

TuMV is noted as a particularly damaging pathogen worldwide, causing diseases in more than 300 mono- and dicotyledonous plant species, including not only economically important vegetable, oilseed, forage, and ornamental crops, but also a wide range of weed species [9,10]. TuMV is transmitted in a non-persistent manner by aphids. Similar to other potyviruses, its genome consists of a single-stranded positive sense RNA molecule of ca. 9800 nucleotides. It contains one open reading frame (ORF) encoding a large polyprotein that is processed to at least 10 final gene products [11]. An additional product P3N-PIPO produced from an alternative ORF, probably by a polymerase slippage mechanism, has been detected, as in other potyviruses [12,13].

The currently known TuMV diversity includes six TuMV genogroups revealed by phylogenetic analysis of numerous TuMV isolates collected around the world: basal-B (*Brassica*), basal-BR (*Brassica*/*Raphanus*), Asian-BR, world-B, Iranian, and Orchis [14,15]. Despite the huge number of sequences accumulated during the recent years, the only genome sequence from oilseed poppy, originating from Poland, was reported as belonging to the world-B group [14].

In the present work, 4 complete TuMV genomes from a cultivated and a wild-growing *Papaver* sp. were characterised, showing their respective affiliation to the world-B and basal-B groups. Moreover, biological assays indicated that intensity of symptoms induced in experimentally inoculated oilseed poppy were correlated with the virus accumulation levels in leaves.

## 2. Material and Methods

### 2.1. Samples

An oilseed poppy plant (*P. somniferum*, labelled as HC9) showing suspicious symptoms (chlorosis, slight deformation of leaf lamina, Supplemental Figure S1) and growing in a commercial field near Hlohovec, western Slovakia, was sampled in May 2016 and subjected to the high throughput sequencing (HTS).

Additionally, a total of 56 oil poppy plants and 10 samples from common poppy (*P. rhoeas*), growing as weeds near cultivated oilseed poppy plants in 5 different locations in Slovakia in early June 2017 (Pezinok, Hlohovec, Malý Šariš, Habovka, Martin) were tested for the presence of TuMV using double-antibody sandwich enzyme-linked immunosorbent assay (DAS-ELISA, Bioreba, Reinach, Switzerland). Two samples from oilseed poppy (MS4 and MS15, both from Malý Šariš) and 1 sample from common poppy (PK1, from Pezinok) testing positive for TuMV were further processed by HTS.

### 2.2. Analysis of the Virome and Determination of the TuMV Full Length Genome Sequences by HTS

Total RNAs from infected poppy leaves were extracted using the NucleoSpin RNA Plant kit (Macherey-Nagel, Duren, Germany) and ribosomal RNA was removed using the Ribo-Zero rRNA Removal Kit (Illumina, San Diego, CA, USA). The rRNA-depleted total RNAs sample was used for double stranded cDNA synthesis using the SuperScript II (Thermo Fisher Scientific, Waltham, MA, USA) kit. The cDNA was then column-purified with the DNA Clean & Concentrator™-5—DNA kit (Zymo Research, Irvine, CA, USA) and quantified with the Qubit 2.0 Fluorometer (Thermo Fisher Scientific, Waltham, MA, USA). The sample was then processed with the transposon-based chemistry library preparation kit (Nextera XT, Illumina, San Diego, CA, USA). Low-cycle PCR and mutual indexing of the fragments was carried out. Fragments were purified with 1.8× AMPure XP beads (BeckmanCoulter, Brea, CA, USA) without size selection. The fragment size structure of the DNA library was assessed using the Agilent 2100 Bioanalyzer (Agilent Technologies, Santa Clara, CA, USA). The equimolar pool of 4 nM DNA libraries was denatured, diluted to 13 pM and sequenced (200-bp paired-end sequencing) on the Illumina MiSeq platform (Illumina, San Diego, CA, USA).

High-quality trimmed reads were used for de novo assembly and contigs aligned to the viral genomes database (ftp://ftp.ncbi.nih.gov/genomes/Viruses/all.fna.tar.gz) using CLC Genomics Workbench 7.5 (Qiagen, Hilden, Germany) and Geneious v.8.1.9. (Biomatters, Auckland, New Zealand) Subsequently, the reads were mapped against the reference TuMV sequence (NC_002509). When necessary, the accuracy of the obtained HTS-based sequences was verified by direct Sanger sequencing of PCR products using primers designed from the HTS-based sequences. In parallel, to assess the intra-isolate sequence heterogeneity, PCR products were cloned into the pGEM-T Easy cloning vector (Promega, Madison, WI, USA) and 6–8 randomly chosen clones were sequenced using universal pUC primers.

The nearly complete nucleotide TuMV sequences reported in this paper (missing 37 nt at the 5′ extremity as compared to the reference NC_002509 genome) have been deposited in the GenBank database under accession numbers MH469724 (PK1), MH469725 (HC9), MH469726 (MS15), and MH469727 (MS04).

Phylogenetic analyses and comparisons with available TuMV sequences (www.ncbi.nlm.nih.gov) were performed using the MEGA v.7 [16] and DnaSP v.5 [17] programs.

Searches for potential recombination events and identification of recombination breakpoints employed seven methods, including RDP, GENECONV, Bootscan, Maxchi, Chimaera, SiScan, and 3SEQ, implemented in the RDP4 v.4.1 software [18] using default settings. Recombination events were noted if supported by at least four different methods (*p*-values < 1.0 × 10^−6^).

### 2.3. Inoculation of Oilseed Poppy and Experimental Plants

The oilseed poppy genotypes ZB24 and ZB25, new-breeding lines of the Slovak origin (National Agriculture and Food Centre, Malý Šariš, Slovakia), were tested for their susceptibility to the TuMV isolates HC9 and PK1 after experimental mechanical inoculation. For the inoculation experiments, homogenous lots of 12 plants were mechanically inoculated at the 3–4 true leaves stage using an inoculum prepared by grinding TuMV-infected leaves 1:10 (*w*/*v*) in 0.1 M sodium phosphate buffer, pH 7.0. *Nicotiana benthamiana*, *Raphanus sativus* cv. Slovana and *R. sativus* subs. Niger cv. Kulatá černá were simultaneously inoculated as controls. Inoculated plants were grown in an insect-proof cultivation room under controlled conditions (14 h light/10 h dark photoperiod, 55 μmol·m^−2^·s^−1^ photon flux density, day/night temperature: 25/18 °C). Development of symptoms was monitored visually and TuMV presence was verified by DAS-ELISA 14 days post inoculation. The experiment was repeated 2 times.

### 2.4. Estimation of Viral Accumulation in Plants

The estimation of viral accumulation in oilseed poppy leaves were performed 14 days post inoculation, using plant issues from second inoculation experiment. The uppermost fully developed leaves were sampled from the plants and equal leaf weight was used for sample preparation. Semiquantitative DAS-ELISA was performed following a previously described protocol [19]. All the samples were homogenised in PBS buffer (1/25 *w*/*v*) containing 0.05% Tween-20 and 2% polyvinylpyrrolidone and subsequently applied to the same ELISA plate, so that absorbance values measured at 405 nm for different genotypes could be compared.

For western blot analysis [20], the crude sap from plant leaves (ground 1:5 in PBS and clarified by 5 min centrifugation at 13,000 g) was mixed 1:1 with Laemmli sample buffer, subjected to SDS-PAGE in 12% gel and stained by Coomassie Brilliant Blue (CBB). Alternatively, the proteins were transferred to a PVDF membrane by a semidry blotting apparatus and detected using polyclonal anti-TuMV antibody (Bioreba, cat. No. 161015)/goat anti-rabbit IgG antibody conjugated with alkaline phosphatase (Sigma, St. Louis, MO, USA) and NBT/BCIP as substrate.

## 3. Results and Discussion

In an effort to assess the presence of viral pathogens naturally infecting poppy, total RNAs isolated from leaves of an oilseed poppy plant showing virus-like symptoms were subjected to HTS. The analysis of the sequence data obtained from this sample (HC9, ca. 2.1 millions of reads of mean length 89.7 nucleotides) enabled us to obtain a large contig of ca. 9 kb, corresponding to TuMV. This was further confirmed by the mapping of individual reads against the NC_002509 reference TuMV sequence, allowing the reconstruction of the nearly complete genome (missing 37 nt at the 5′ end as compared to NC_002509) with a mean coverage of 483×. No other viral agent was identified in the HC9 sample.

This discovery prompted us to perform a screening for TuMV presence in a larger set of 66 samples of *Papaver somniferum* and *P. rhoeas* from 5 different locations in Slovakia. DAS-ELISA revealed TuMV infection in 2 out of 56 oilseed poppy plants and in 1 out of 10 common poppy plants. These samples, labelled as MS4 and MS15 (both from *P. somniferum* grown in Malý Šariš) and PK1 (from *P. rhoeas* found in Pezinok), were further analysed using HTS.

De novo assembly of high-quality reads (between 2.1 and 3.1 million depending on the sample) from MS4, MS15, and PK1 resulted in the assembly of nearly complete TuMV genomes (mean coverage 210–709×). While only TuMV was identified in the MS4 and MS15 samples, the PK1 sample was found to be co-infected with *Cucumber mosaic virus* (CMV).

In accordance with typical potyvirus genomic organization [11,21], a large ORF (9489–9495 nts in length) was identified in all 4 isolates, encoding a polyprotein of 3162 aa (PK1) or 3164 aa (HC9, MS4, MS15) with an estimated MW of 357.30–357.45 kDa, respectively (http://web.expasy.org/protparam/). A two-amino acid deletion at position 39–40 of the capsid protein (aa 2915–2916 based on the reference NC_002509 polyprotein) is responsible for a shorter polyprotein of PK1. Nine putative cleavage sites were identified in all polyproteins. The PIPO ORF [12] was identified in silico downstream of the highly conserved motif GA6 (nt position 3041–3047). While PIPO of the PK1 isolate encodes deduced product of 60 aa, that identified in HC9, MS04, and MS15 encodes a longer product of 69 aa.

All potyvirus motifs involved in the virus infectious cycle were perfectly conserved among the four Slovak TuMV poppy isolates at the position previously indicated [22]. The conservative motifs essential for aphid transmission of potyviruses (KITC_414–417_, PTK_672–674_, DAG_2882–2884_) were found in their polyprotein sequences at the expected positions.

**Figure 1:**
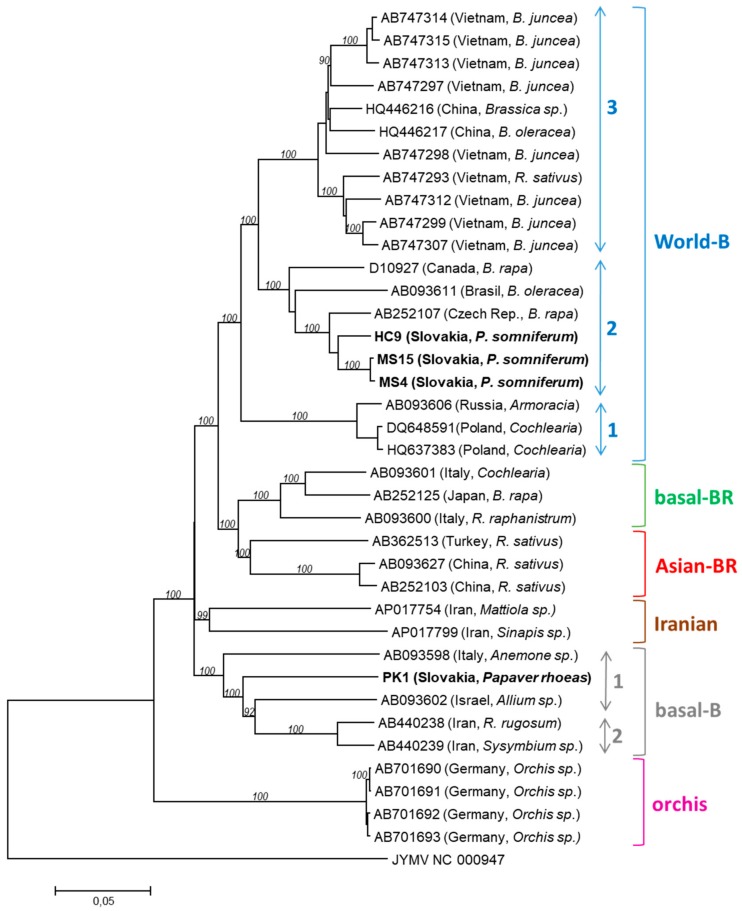
Neighbour-joining tree inferred from the nearly complete TuMV genomes including those determined in this work (in bold). Non-recombinant database sequences were used (according to [14,24]), identified by acc. number, original host and geographical location. Bootstrap percentages (>70%) based on 1000 pseudoreplicates are indicated at a given node. The scale bar indicates 0.05 substitutions per site. The genomic sequence of Japanese yam mosaic virus (JYMV) was used as an outgroup.

Based on a Blast search, the closest relatives of the PK1 isolate are basal-B group isolates from Greece (GRC45, GRC70) and Turkey (TUR59, TUR60, TUR61), but PK1 divergence is significant, as it shows only ca. 89% nt identity with them. PK1 thus appears as a divergent isolate of the basal-B genogroup. On the contrary, MS04 and MS15 (having 99.6% mutual nt identity) were 99% identical to the Croatian CRO184A isolate while HC9 was ca. 97% identical to the CZE5, POL1, and POL2 isolates from the Czech Republic and Poland, respectively, all of which belong to the word-B group (subgroup 2). The affiliation of Slovak poppy isolates was further confirmed by a phylogenetic analysis performed using a complete database of non-recombinant sequences belonging to the 6 recognised TuMV genogroups [14,15] (Figure 1).

RNA recombination is one of the driving forces shaping the genetic variability in TuMV, with various recombination events described, including both intra- and inter-lineage recombinations [23,24,25,26,27]. The four Slovak isolates were assessed for possible evidence of recombination using a set of 33 non-recombinant TuMV isolates [14,15] (accession numbers listed in Figure 1). The RDP4 analysis [18] revealed a recombination event in the genome of the PK1 isolate, with breakpoints in the HC-Pro gene (nt positions 1195–2317). This event was supported by 5 programs of the RDP4 package (for details see Supplemental Table S1). Using a Blast search, the closest potential donor sequence (indicating the lineage that was likely to have provided this region of the recombinant genome) was found to be the Turkish AP017736 isolate from *Raphanus raphanistrum* with 91% nucleotide identity. Although a number of recombination events affecting TuMV have been described [14,15,23,24,25,26,27], the recombination pattern and breakpoints identified in the PK1 genome had not been previously reported.

The genome of the other three Slovak isolates (HC9, MS4, and MS15) did not reveal statistically supported evidence for recombination. However, HC9 was tentatively identified as a minor parent of the Czech AB252107 isolate and MS15 as a tentative minor parent involved in a recombination event affecting the 3′ region of the genome of several Vietnamese and Chinese isolates from the world-B group (AB747293, AB747297, AB747307, AB747312-15, HQ446216-17, Figure 1), all undergoing a previously overlooked intra-lineage recombination (supported by 6 algorithms, Supplementary Table S1). Our results thus further highlight the important role of recombination in the evolutionary history of TuMV.

Despite the importance of TUMV and its negative effect to the production of many crops, mainly the studies focusing on *Brassicaceae* were reported [28,29,30,31,32]. On the contrary, there is no data about the TuMV/oilseed poppy relationship [6]. The susceptibility of two oilseed poppy genotypes, ZB24 and ZB25, was therefore evaluated by experimental mechanical inoculation with TuMV isolates PK1 and HC-9, belonging respectively to the world-B and basal-B molecular clades.

As only TuMV infection was revealed by HTS analysis of the HC9 sample, the original leaf sample, stored at −80 °C, was used for these experimental inoculations, providing reassurance on the purity of the inoculum. In parallel, since the original PK1 sample was found coinfected with CMV, to ensure a single TuMV infection, the plant sap from original host plant was highly diluted (1/150, *w*/*v*) and a set of *Nicotiana benthamiana* plants were mechanically inoculated. These tobacco plants were then individually tested for TuMV and CMV presence. An unambiguously TuMV singly-infected plant, repeatedly tested CMV-negative using ELISA, RT-PCR, and back inoculation to *Cucumis sativus* was used as the source of PK1 TuMV isolate (details of CMV detection methods used available upon request).

The natural viral isolates consist of a cloud of closely related viral genomes, called quasispecies, and this mutant spectra and not individual genomes are the target of evolutionary events [33]. This gentle sequence heterogeneity is usually overlooked in standard sequencing efforts. In this work, an “isolate” refers to the determined single consensus dominant sequence, further used for comparisons of isolates and phylogenetic purposes.

The intra-isolate sequence homogeneity estimated from HTS reads and from sequencing of individual PCR-derived clones suggested the presence of unique TuMV population in each sample. Therefore, to not impose additional selection pressure to TuMV population, a passage through a local lesion host was omitted. However, we cannot exclude the possibility of a mixed infection of TuMV population by a contaminant TuMV variant. If any, such a low-frequency heterogeneity would not have an impact on the biological or molecular data obtained.

**Figure 2:**
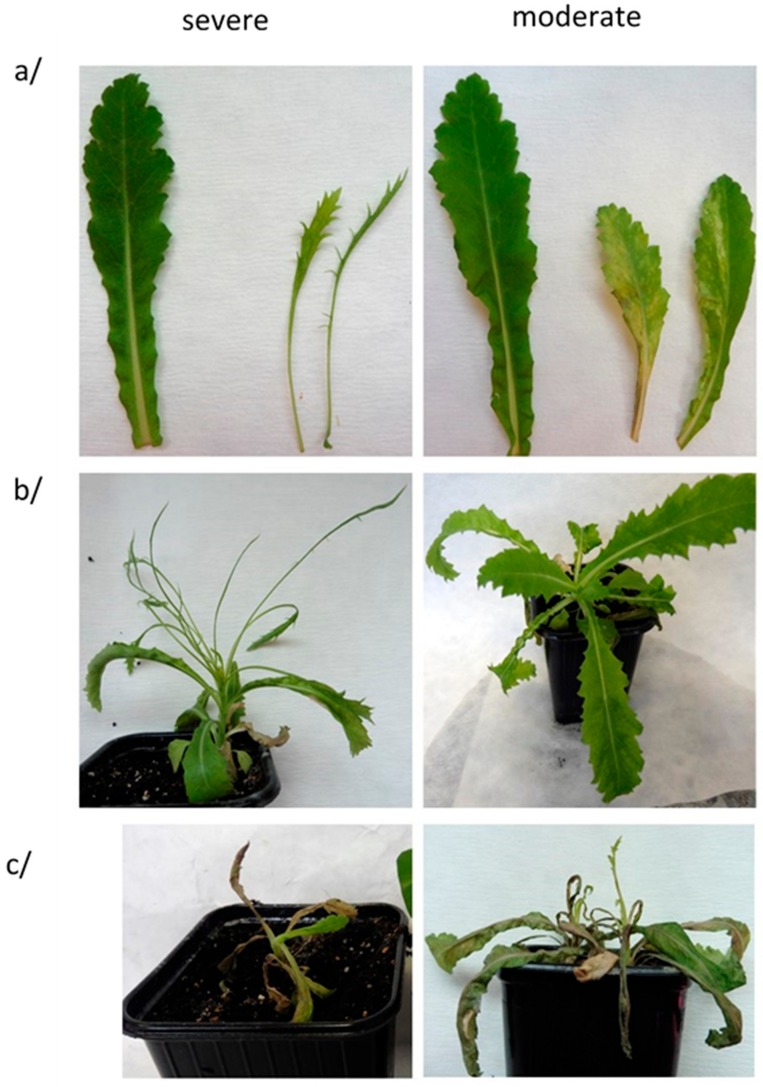
Two symptom types induced by mechanical inoculation of oilseed poppy ZB24 plants. (**a**) detail of leaf symptoms and comparison with a healthy leaf, (**b**) infected oilseed poppy plant 14 days p.i., (**c**) infected oilseed poppy plant 35 days p.i.

Both TuMV isolates could be mechanically transmitted to oilseed poppy plants with 40–66% efficacy (Table 1) as proved by DAS-ELISA. None of the symptomless plants tested TuMV-positive, showing a clear correlation between symptoms expression and virus presence as detected by DAS-ELISA. Different inoculum sources were used for these biological assays, likely resulting in different virus titers (original infected oilseed poppy leaves for HC9 or leaves of experimentally infected *N. benthamiana* for PK1, respectively), therefore no effort was made to compare the intensity of the symptoms induced by the two isolates. Notwithstanding, in all cases, two types of symptoms were observed (Table 1, Figure 2). One type of symptom consisted of chlorotic patterns and dark green spots on the leaf lamina (termed as “moderate”). The second type of symptom (“severe”) consisted of shoestringing, an extreme leaf deformation with loss of the limb part of the leaves (Figure 2). Irrespective of the symptomatology type, subsequent systemic necrosis was observed, leading to the stunting, wilting, and finally dying of positive plants 35–45 days post-inoculation. The experimental inoculation of *N. benthamiana* plants lead to 100% infection, with both TuMV isolates inducing leaf deformation, mosaics, and plant stunting. On the contrary, only one out of 40 *R. sativus* inoculated plants ended up infected, suggesting that this species may not be adapted to the study of Slovak TuMV isolates from poppy.

**Figure 3:**
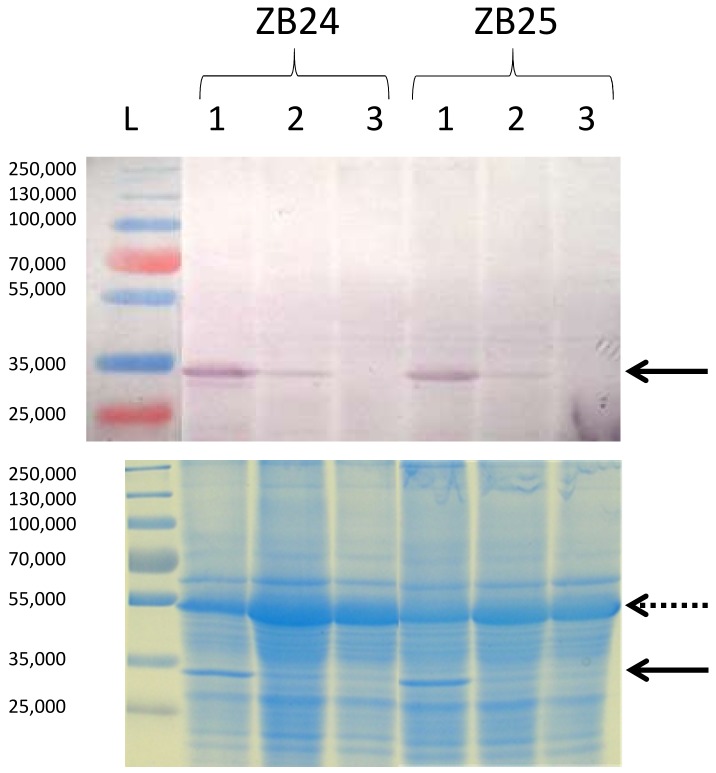
Immunoblot analysis of crude plant sap obtained from TuMV-infected ZB24 and ZB25 oilseed poppy leaves with different symptomatology 14 days p.i. (upper panel). A CBB-stained gel is in the lower panel. L—PageRuler Plus Prestained Protein Ladder (Thermo Scientific), 1—plant with severe symptoms, 2—plant with moderate symptoms, 3—uninfected control plant. Arrows indicate the position of TuMV CP. Dotted arrow shows ribulose-1,5-bisphosphate carboxylase/oxygenase large chain serving as loading control.

The symptoms and their development are the result of a complex plant–virus interplay [34]. Moderate and severe symptoms were observed in both oilseed poppy genotypes, irrespective of the TuMV isolate used, suggesting that this is apparently not an isolate- or host genotype-specific phenomenon. To investigate it further, the accumulation of viral CP was evaluated in symptomatic leaves showing either the moderate or the severe symptoms. Semi-quantitative DAS-ELISA provided approximately 2.2–3.1 times higher OD values for samples prepared from leaves showing severe symptoms (Table 1). This correlation of TuMV CP accumulation with the symptoms type was confirmed by immunoblotting (Figure 3). These results indicate that the intensity of symptoms was positively correlated with virus accumulation in the leaf.

Possible hypotheses to explain the observed differences in symptomatology of oilseed poppy plants of the same genotype and inoculated with the same TuMV isolate could be a slightly uneven initial inoculum load [35] and/or differences in the physiological states of the young inoculated plants, despite the precautions taken to standardise the experiment. Although the experimental mechanical transmission of TuMV did not mimic the natural inoculation of the virus by its aphid vectors, our results suggest that natural TuMV infection of young oilseed poppy plants could also result in their death by systemic necrosis under field conditions.

In conclusion, the present work extends our knowledge on the host range of TuMV, showing world-B and basal-B TuMV isolates are able to naturally infect both cultivated and weed *Papaver* sp. and to cause significant symptoms in these hosts.

## Figures and Tables

**Table 1:** Evaluation of plant susceptibility to the experimental TuMV infection.

	*Papaver somniferum* ZB24		*Papaver somniferum* ZB25		*N. benthamiana* ^a^	*R. sativus* cv. Slovana ^a^	*R. sativus* cv. Kulatá černá ^a^
Isolate	Experiment 1	Experiment 2	Experiment 1	Experiment 2			
HC9	12/7 ^b^ (2 ^m^ + 5 ^s^)	12/5 (2 ^m^ + 3 ^s^) m = 0.552 ± 0.045 *s = 1.711 ± 0.129	10/4 (1 ^m^ + 3 ^s^)	12/6 (2 ^m^ + 4 ^s^) m = 0.333 ± 0.049 s = 0.799 ± 0.184	10/10	20/0	20/0
PK1	12/8 (4 ^m^ + 4 ^s^)	11/6 (4 ^m^ + 2 ^s^) m = 0.652 ± 0.034 s = 1.589 ± 0.108	12/7 (3 ^m^ + 4 ^s^)	11/6 (3 ^m^ + 3 ^s^) m = 0.417 ± 0.057 s = 0.983 ± 0.139	10/10	20/1	20/0

^a^—merged results from 2 experiments, ^b^—nb of plants/nb of TuMV infected plants as detected by DAS-ELISA, ^m^—moderate symptoms, ^s^—severe symptoms, *—absorbance measured at 405 nm after 1 h (mean ± SD).

## Supplementary Materials

The following are available online at http://www.mdpi.com/1999-4915/10/8/430/s1, Figure S1: Leaf symptoms on the original HC9 oilseed poppy plant (collected on June from a developed plant early after flowering), Table S1: The most likely recombination event in the sequences of TuMV isolates.

## References

[1] Baroš S., Karšayová B., Jomová K., Gáspár A., Valko M. (2012). Free radical scavenging capacity of *Papaver somniferum* L. and determination of pharmacologically active alkaloids using capillary electrophoresis. J. Microbiol. Biotechnol. Food Sci..

[2] Lančaričová A., Havrlentová M., Muchová D., Bednárová A. (2016). Oil content and fatty acids composition of poppy seeds cultivated in two localities of Slovakia. Agriculture (Polnohospodárstvo).

[3] Krošlák E., Maliar T., Nemeček P., Viskupičová J., Maliarová M., Havrlentová M., Kraic J. (2017). Antioxidant and proteinase inhibitory activities of selected poppy (*Papaver somniferum* L.) genotypes. Chem. Biodivers..

[4] Lutman P.J.W., Cussans G.W., Wright K.J., Wilson B.J., Lawson H.M. (2002). The persistence of seeds of 16 weed species over six years in two arable fields. Weed Res..

[5] Brunt A.A., Crabtree K., Dallwitz M.J., Gibbs A.J., Watson L. (1996). Turnip Mosaic Potyvirus. Viruses of Plants.

[6] Kubelková D., Špak J. (1999). Virus diseases of poppy (*Papaver somniferum* L.) and some other species of the Papaveraceae family. Plant Prot. Sci..

[7] Tang J., Lebas B., Liefting L., Veerakone S., Wei T., Ward L. (2016). Opium poppy mosaic virus, a new umbravirus isolated from *Papaver somniferum* in New Zealand. Arch. Virol..

[8] Srivastava A., Kumar S., Jaidi M., Raj S.K., Shukla S.K. (2016). First Report of Tomato leaf curl New Delhi virus on Opium Poppy (*Papaver somniferum*) in India. Plant Dis..

[9] Jenner C.E., Walsh J.A. (1996). Pathotypic variation in *Turnip mosaic virus* with special reference to European isolates. Plant Pathol..

[10] Walsh J.A., Jenner C.E. (2002). *Turnip mosaic virus* and the quest for durable resistance. Mol. Plant Pathol..

[11] Ohshima K., Tanaka M., Sako N. (1996). The complete nucleotide sequence of *Turnip mosaic virus* RNA Japanese strain. Arch. Virol..

[12] Chung B.Y., Miller W.A., Atkins J.F., Firth A.E. (2008). An overlapping essential gene in the Potyviridae. Proc. Natl. Acad. Sci. USA.

[13] White K.A. (2015). The polymerase slips and PIPO exists. EMBO Rep..

[14] Nguyen H.D., Tomitaka Y., Ho S.Y.W., Duchêne S., Vetten H.-J., Lesemann D., Walsh J.A., Gibbs A.J., Ohshima K. (2013). Turnip mosaic potyvirus probably first spread to Eurasian brassica crops from wild orchids about 1000 years ago. PLoS ONE.

[15] Yasaka R., Fukagawa H., Ikematsu M., Soda H., Korkmaz S., Golnaraghi A., Katis N., Ho S.Y.W., Gibbs A.J., Ohshima K. (2017). The Timescale of Emergence and Spread of Turnip Mosaic Potyvirus. Sci. Rep..

[16] Kumar S., Stecher G., Tamura K. (2016). MEGA7: Molecular Evolutionary Genetics Analysis version 7.0 for bigger datasets. Mol. Biol. Evol..

[17] Librado P., Rozas J. (2009). DnaSP v5: A software for comprehensive analysis of DNA polymorphism data. Bioinformatics.

[18] Martin D.P., Murrell B., Golden M., Khoosal A., Muhire B. (2015). RDP4: Detection and analysis of recombination patterns in virus genomes. Virus Evol..

[19] Glasa M., Labonne G., Quiot J.-B. (2003). Effect of temperature on Plum pox virus infection. Acta Virol..

[20] Šubr Z., Glasa M. (1999). Plum pox virus capsid protein mobility in SDS-polyacrylamide gel electrophoresis. Acta Virol..

[21] Adams M.J., Antoniw J.F., Fauquet C.M. (2005). Molecular criteria for genus and species discrimination within the family Potyviridae. Arch. Virol..

[22] Zhu F., Sun Y., Wang Y., Pan H., Wang F., Zhang X., Zhang Y., Liu J. (2016). Molecular Characterization of the Complete Genome of Three Basal-BR Isolates of *Turnip mosaic virus* Infecting *Raphanus sativus* in China. Int. J. Mol. Sci..

[23] Ohshima K., Yamaguchi Y., Hirota R., Hamamoto T., Tomimura K., Tan Z., Sano T., Azuhata F., Walsh J.A., Fletcher J. (2002). Molecular evolution of *Turnip mosaic virus*: Evidence of host adaptation; genetic recombination and geographical spread. J. Gen. Virol..

[24] Ohshima K., Tomitaka Y., Wood J.T., Minematsu Y., Kajiyama H., Tomimura K., Gibbs A.J. (2007). Patterns of recombination in *Turnip mosaic virus* genomic sequences indicate hotspots of recombination. J. Gen. Virol..

[25] Nguyen H.D., Tran H.T.N., Ohshima K. (2013). Genetic variation of the *Turnip mosaic virus* population of Vietnam: A case study of founder; regional and local influences. Virus Res..

[26] Tomimura K., Gibbs A.J., Jenner C.E., Walsh J.A., Ohshima K. (2003). The phylogeny of *Turnip mosaic virus*; comparisons of 38 genomic sequences reveal a Eurasian origin and a recent ‘emergence’ in East Asia. Mol. Ecol..

[27] Wang H.Y., Liu J.L., Gao R., Chen J., Shao Y.H., Li X.D. (2009). Complete genomic sequence analyses of *Turnip mosaic virus* basal-BR isolates from China. Virus Genes.

[28] Suehiro N., Natsuaki T., Watanabe T., Okuda S. (2004). An important determinant of the ability of *Turnip mosaic virus* to infect *Brassica* spp. and/or *Raphanus sativus* is in its P3 protein. J. Gen. Virol..

[29] Coutts B.A., Walsh J.A., Jones R.A.C. (2007). Evaluation of resistance to *Turnip mosaic virus* in Australian *Brassica napus* genotypes. Aust. J. Agric. Res..

[30] Kehoe M.A., Coutts B.A., Jones R.A.C. (2010). Resistance phenotypes in diverse accessions; breeding lines; and cultivars of three mustard species inoculated with *Turnip mosaic virus*. Plant Dis..

[31] Nyalugwe E.P., Barbetti M.J., Jones R.A.C. (2014). Preliminary studies on resistance phenotypes to *Turnip mosaic virus* in *Brassica napus* and *B. carinata* from different continents and effects of temperature on their expression. Eur. J. Plant Pathol..

[32] Guerret M.G.L., Nyalugwe E.P., Maina S., Barbetti M.J., van Leur J.A.G., Jones R.A.C. (2017). Properties of a *Turnip mosaic virus* (TuMV) strain that breaks TuMV resistances in *Brassica napus*. Plant Dis..

[33] Domingo E., Sheldon J., Perales C. (2012). Viral quasispecies evolution. Microbiol. Mol. Biol. Rev..

[34] Garcia J.A., Pallas V. (2015). Viral factors involved in plant pathogenesis. Curr. Opin. Virol..

[35] Rodrigo G., Zwart M.P., Elena S.F. (2014). Onset of virus systemic infection in plants is determined by speed of cell-to-cell movement and number of primary infection foci. J. R. Soc. Interface.

